# Unravelling the electronic structure and dynamics of an isolated molecular rotary motor in the gas-phase†
†Electronic supplementary information (ESI) available: Details of molecular motor synthesis and NMR data; geometrical data of optimised structures used in computational chemistry calculations; natural orbitals of the active space used in state-averaged CASSCF calculations. See DOI: 10.1039/c7sc01997a



**DOI:** 10.1039/c7sc01997a

**Published:** 2017-06-27

**Authors:** Reece Beekmeyer, Michael A. Parkes, Luke Ridgwell, Jamie W. Riley, Jiawen Chen, Ben L. Feringa, Andrew Kerridge, Helen H. Fielding

**Affiliations:** a Department of Chemistry , University College London , 20 Gordon Street , London , WC1H 0AJ , UK . Email: h.h.fielding@ucl.ac.uk; b Department of Chemistry , Lancaster University , Lancaster , LA1 4YB , UK; c Stratingh Institute for Chemistry , University of Groningen , Nijenborgh 4 , 9747 AG Groningen , The Netherlands

## Abstract

Light-driven molecular motors derived from chiral overcrowded alkenes are an important class of compounds in which sequential photochemical and thermal rearrangements result in unidirectional rotation of one part of the molecule with respect to another. Here, we employ anion photoelectron spectroscopy to probe the electronic structure and dynamics of a unidirectional molecular rotary motor anion in the gas-phase and quantum chemistry calculations to guide the interpretation of our results. We find that following photoexcitation of the first electronically excited state, the molecule rotates around its axle and some population remains on the excited potential energy surface and some population undergoes internal conversion back to the electronic ground state. These observations are similar to those observed in time-resolved measurements of rotary molecular motors in solution. This work demonstrates the potential of anion photoelectron spectroscopy for studying the electronic structure and dynamics of molecular motors in the gas-phase, provides important benchmarks for theory and improves our fundamental understanding of light-activated molecular rotary motors, which can be used to inform the design of new photoactivated nanoscale devices.

## Abstract 2

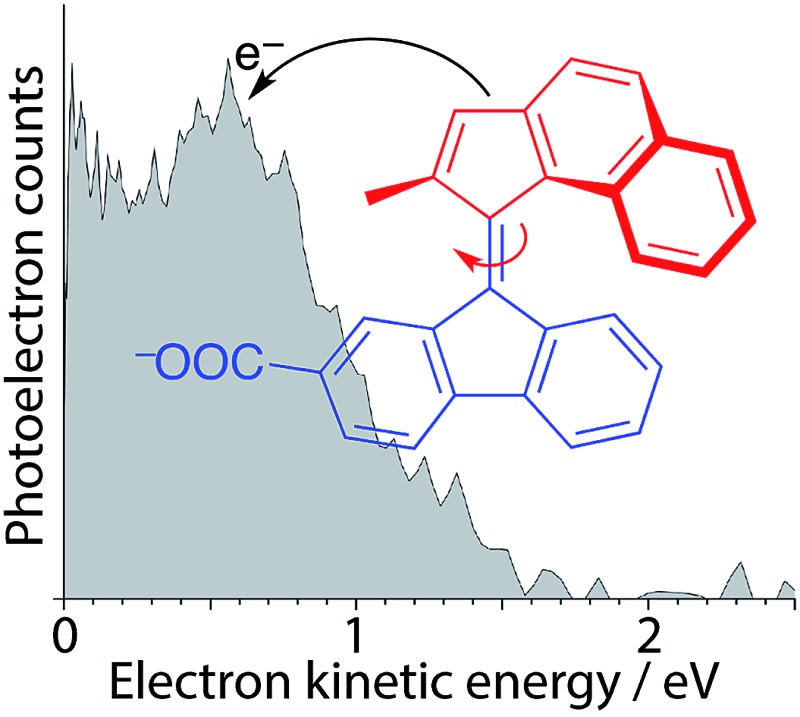
Anion photoelectron spectroscopy and quantum chemistry calculations are employed to probe the electronic structure and dynamics of a unidirectional molecular rotary motor anion in the gas-phase.

## 1 Introduction

Molecular machines are molecules that are able to absorb energy and convert it into non-Brownian motion.^1–10^ Their significance has been acknowledged by the award of the 2016 Chemistry Nobel Prize to Jean-Pierre Sauvage, Sir J. Fraser Stoddart and Bernard L. Feringa for “the design and synthesis of molecular machines”. An important class of molecular machines are light-driven molecular rotary motors, first developed by Feringa and co-workers in 1999.^11^ These rotary molecular motors are chiral overcrowded alkenes in which one part of the molecule (rotor) undergoes unidirectional rotary motion with respect to another part of the molecule (stator) around a central carbon–carbon double bond (axle).

**Fig. 1:**
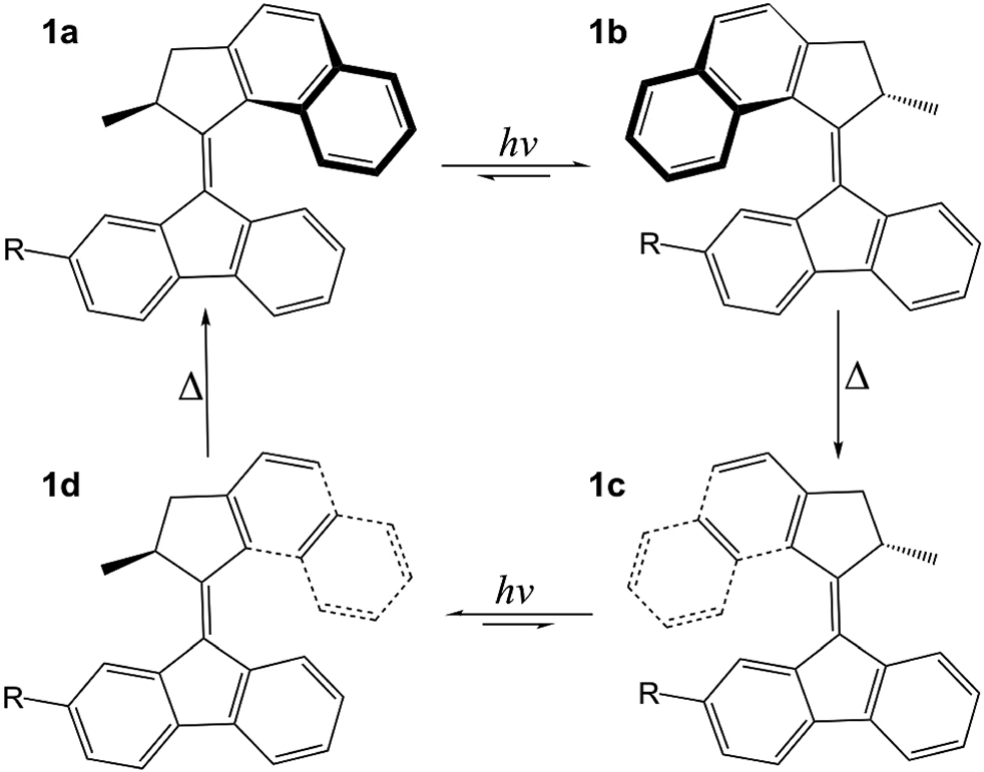
Structure of the molecular motor employed in these studies (R = COO^–^). The initial steps of the photocycle are photochemical excitation, isomerisation and internal conversion back to the ground state (**1a–1b**). This is followed by thermal helix inversion on the ground state (**1b–1c**), a second photochemical excitation, isomerisation and internal conversion (**1c–1d**) and, finally, a second thermal helix inversion on the ground state to form the original isomer (**1d–1a**).

The rotary cycle of a fluorene-based molecular motor is illustrated in Fig. 1. In its lowest energy conformation (**1a**), the methyl substituent at the stereogenic centre adopts an axial orientation in which steric hindrance is minimised. Following absorption of ultraviolet (UV) light, excited state photoisomerisation causes the molecule to rotate around the axle (**1b**) and in this conformation the methyl substituent is forced to adopt an equatorial orientation. This step is sometimes referred to as the photochemical power stroke and determines the photochemical conversion efficiency of the motor. Following relaxation back to the ground electronic state, increased steric interactions between the rotor and the stator create tension in the molecule that is subsequently released as the rotor and stator blades snap over one another in a thermal helix inversion (**1b–1c**) and the methyl group re-adopts its thermodynamically favourable axial orientation. Importantly, the steric barrier to reverse rotation on the ground electronic state locks the molecular motor in this conformation until it can absorb another photon. A full unidirectional rotary cycle is then completed following a second UV photoisomerisation and ground-state thermal helix inversion (**1c–1d–1a**).

The rate determining step in the rotary cycle is the thermal helix inversion on the ground electronic state. This has been confirmed by time-resolved transient absorption spectroscopy measurements of molecular motors dissolved in hexane that found the light-induced isomerisation step to be completed in 20 ps.^12^ A great deal of synthetic effort has been focussed on designing and synthesising rotary motors with lower barriers to thermal helix inversion and higher rotation frequencies;^13–21^ however, there has been relatively little interest in optimising the photochemical power stroke. Meech and co-workers reported a series of very detailed time-resolved fluorescence and transient absorption spectroscopy measurements probing the initial photoisomerisation step of rotary motors dissolved in a range of solvents.^22,23^ They found that following photoexcitation, rapid structural relaxation out of the Franck–Condon region occurred on a timescale of a few 100 fs to populate a ‘dark state’. This ‘dark state’ remained coupled to the Franck–Condon region and relaxed through a conical intersection back to the ground state on a picosecond timescale, completing the light-induced isomerisation step. They found that attaching electron withdrawing and electron donating substituents in direct conjugation with the central carbon–carbon double bond modified the structure and energy of the ‘dark state’ and thus the efficiency of operation of these molecular motors, without affecting the rotation frequency. Recently, Amirjalayer *et al.* reported a detailed time-resolved infrared spectroscopy study, supported by quantum mechanical calculations, which hypothesised that electronic relaxation proceeds on the potential energy surfaces of two different electronic states and thus indicates that the efficiency of the motor is determined by the conical intersection between the ‘dark state’ and the ground state.^24^

From a theoretical perspective, there have been numerous studies of molecular rotary motors aimed at understanding how both the molecular structure and electronic structure influence the excited state and ground state dynamics. Kazaryan *et al.* used ensemble DFT (density functional theory) and OM2/MRCI molecular dynamics calculations to explore the potential energy surface of a fluorene based motor.^25–27^ They found barriers to rotation along the ground electronic state, S_0_, as well as the excitation energy to the first excited state, S_1_, using ensemble DFT and PM3 methods and, in agreement with the later experimental observations,^22,23^ they predicted that a conical intersection between the S_0_ and S_1_ states played a key role in the light-induced isomerisation step. Their MD simulations found an average excited state lifetime of 1.4 ps. Torras *et al.* employed QM/MM (quantum-mechanics/molecular mechanics) calculations to investigate another fluorene based overcrowded chiral alkene molecular motor and its rotation.^28^ They optimised structures using unrestricted DFT and the perturbative MP2 method and included solvent effects in their QM/MM calculations. They calculated barriers to rotation which were comparable to experiment and also found that the hybridisation of the ethylene axle evolved from being sp^2^ to sp^3^ and back again, as the motor rotated, to minimise steric repulsions. CASSCF (complete active space self consistent field) calculations were carried out by Amatatsu using a 2-orbital, 2-electron active space.^29,30^ Again it was found that a conical intersection between the S_1_ and S_0_ states was a key factor in the light-induced isomerisation step. Recently, Pang *et al.* have reported the results of trajectory surface-hopping dynamics at the semi-empirical OM2/MRCI level investigating the photoisomerisation dynamics;^31^ they found timescales in reasonable agreement with those measured by Meech and coworkers,^22,23^ although they suggest that the ‘dark state’ is not a separate electronic state, as suggested by Amirjalayer *et al.*,^24^ but a ‘dark region’ of the S_1_ state.

To gain a complete understanding of the intrinsic dynamics of a molecular rotary motor requires gas-phase measurements of isolated motor molecules, free from interactions with solvent molecules. Although weakly interacting solvents are generally considered to have little influence on the shapes of molecular potential energy surfaces, differential solvation of electronic states can modify the structure and energy of conical intersections. Solvent molecules also provide an effective sink for vibrational energy relaxation, so they may modify the dynamics on the potential energy surfaces. However, there have not been any reports of gas-phase measurements of the electronic structure and dynamics of molecular rotary motors to date, most likely due to the difficulties associated with generating molecular beams of such large molecules. Electrospray ionisation has proved a very effective method for transferring large molecules into the gas-phase in deprotonated anionic or protonated cationic forms. Here, we employ electrospray-ionisation to transfer a molecular rotary motor into the gas-phase, using a carboxylic acid group attached to the stator to provide a spectator group that is easy to deprotonate. We then use anion photoelectron spectroscopy to measure the electronic structure and dynamics of the molecular rotary motor and high-level CASSCF and MS-CASPT2 (multiconfigurational second-order perturbation theory) calculations to guide the interpretation of our experimental measurements. Interestingly, we find that the initial dynamics in the gas-phase are similar to those reported in solution:^22–24^ they involve relaxation away from the Franck–Condon region to a rotated conformer on the excited state and internal conversion back to the ground state. Gas-phase studies such as these, alongside solution phase studies, have the potential to improve our fundamental understanding of light-activated molecular rotary motors and inform the design of photoactivated nanoscale devices.

## 2 Methodology

### 2.1 Synthesis

The preparation of **1a** (R = COOH) is based on the route reported for **1a** (R = H) and the procedures and characterisation are described in the ESI.†

### 2.2 Anion photoelectron spectroscopy

Photoelectron spectra were recorded using our anion photoelectron imaging apparatus.^32–39^ Briefly, deprotonated molecular motor anions were generated by electrospray ionisation of solutions of **1a** (R = COOH) in methanol with a couple of drops of ammonia added. The anions, **1a** (R = COO^–^), were mass selected by a quadrupole and passed into a collision cell, which also acted as a hexapole ion trap to generate packets of anions at a frequency of 20 Hz to match the repetition rate of the laser system. The ions were then transported *via* a potential switch to the interaction region of collinear velocity map imaging optics, where they interacted with nanosecond laser pulses with wavelengths in the range 320–230 nm. Photoelectrons generated in the interaction region were accelerated towards a position sensitive detector consisting of a stack of multichannel plates, a phosphor screen and a CCD camera. Laser-only images were subtracted from images recorded following the interaction of laser light with the anions, to eliminate background counts arising from scattered light and ionisation of residual gas. The photoelectron images were inverted using the pBASEX method^40^ and the energy scale was calibrated by recording the photoelectron spectrum of I^–^. The energy resolution is around 5% and the error in electron kinetic energy is ±0.05 eV.

### 2.3 Computational

Ground-state minimum energy geometries of the anions **1a–1d** (R = COO^–^) and their corresponding neutral radicals were optimised at the B3LYP^41–44^ level using the 6-311G++(2df,2pd) basis.

Vertical excitation energies (VEEs) of the first few singlet excited states of anions **1a–1d** (R = COO^–^) were calculated at DFT-optimised geometries using the state-averaged complete active space self consistent field (SA-CASSCF) method^45^ with equal weighting given to the first four states (SA(4)). The active space consisted of six pairs of π and π* orbitals (12, 12) on the stator, rotor and axle, selected by inspection of the natural orbital occupations (see ESI†). Orbitals localised predominantly on the carboxylic acid group were excluded, since this group was included for experimental reasons and does not contribute significantly to the photochemistry (Section 3.2).

We used the atomic natural orbital (ANO-L) basis set^46^ of polarised triple-zeta (double-zeta for hydrogen) quality (TZVP (14s9p4d3f)/[4s2p2d1f] for carbon and oxygen, DZVP (8s4p3d)/[2s1p] for hydrogen). To reduce computational time, we used the Cholesky decomposition^47^ with the default parameters for computation of two-electron integrals.

To obtain more accurate VEEs, dynamic correlation was included by using second order pertubation theory (PT2) with the multi state formalism (MS-CASPT2),^48^ with the SA(4)-CASSCF wave functions as a reference. We used the standard value of 0.25 for the ionisation potential-electron affinity (IPEA) shift as this has been shown to give more accurate dissociation and excitation energies.^49^ To minimise the effect of intruder states, an imaginary level shift of 0.2 a.u. was used.^50^

Vertical detachment energies (VDEs) of **1a–1d** (R = COO^–^) were calculated as the differences between the MS-CASPT2 energies of the anions and their corresponding neutral radicals, at the DFT optimised geometries of the anions. Adiabatic detachment energies (ADEs) of **1a** and **1b** (R = COO^–^) were calculated as the energy differences between the MS-CASPT2 energies of the DFT optimised geometries of the anions and the MS-CASPT2 energies of the DFT optimised geometries of the corresponding neutral radicals.

The DFT calculations were performed using the Gaussian 09 suite of programs.^51^ SA-CASSCF and MS-CASPT2 calculations were performed using the Molcas 8.0 code.^52–54^

## 3 Results

### 3.1 Photoelectron spectra

**Fig. 2:**
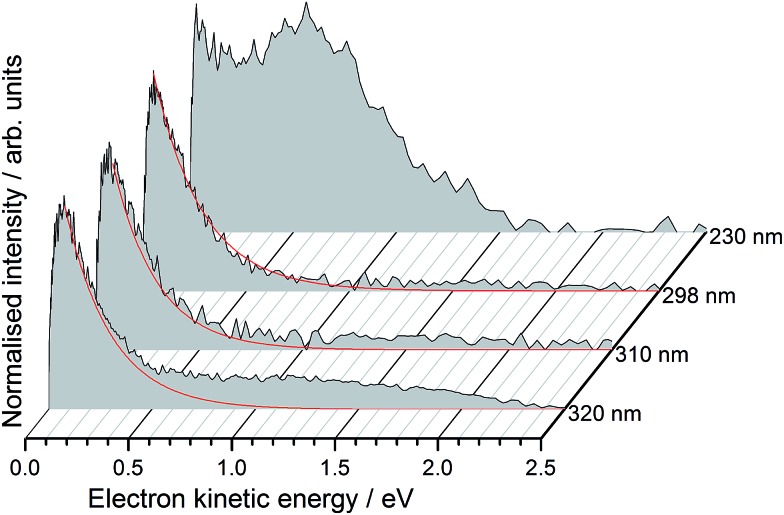
Photoelectron spectra of deprotonated molecular motor **1a** (R = COO^–^) recorded at 320 nm (3.87 eV), 310 nm (4.00 eV), 298 nm (4.16 eV) and 230 nm (5.39 eV), plotted as a function of photoelectron kinetic energy. The solid red lines are exponential fits representing thermionic emission (see text).

Photoelectron spectra of deprotonated molecular motor **1a** (R = COO^–^) were recorded as a function of electron kinetic energy (eKE) and are presented in Fig. 2.

All the photoelectron spectra have an intense, low eKE feature that decays exponentially with increasing eKE, characteristic of thermionic emission from the hot ground state of the molecular motor anion. For the 320–398 nm spectra, the low eKE distributions can be fit to *I*(eKE) = *I*_0_ exp(–eKE/*k*_B_ *T*) to obtain an estimate of the vibrational temperature of the ground state of the anion formed after internal conversion. We found *k*_B_ *T* ≈ 0.2 eV. Although the value of *k*_B_ *T* is not particularly meaningful because there are competing decay mechanisms, such as autodetachment, the observation of thermionic emission is very significant because it shows that internal conversion back to the ground state occurs, following excitation in the range 320–230 nm.

In addition to the exponentially decaying low eKE feature, the 230 nm (5.38 eV) photoelectron spectrum also has a broad, unresolved feature centered around 0.60 eV, corresponding to an electron binding energy, eBE = *hν* – eKE ≈ 4.8 eV. If these photoelectrons arise from direct detachment from the ground electronic state of the anion, the eBE of the peak maximum would correspond approximately to the VDE, although we note that the VDE and maximum may be offset in unresolved photodetachment spectra of relatively large molecular anions.^55^ Such a value for the VDE (4.8 eV) would also explain why direct detachment is not observed at the three lower photon energies (3.87–4.16 eV) used in this work.

Interestingly, there are also weak, continuous photoelectron distributions in the range 1.0–2.0 eV eKE, most noticeably in the 320 nm photoelectron spectrum. We attribute these to multiphoton processes or loss of CO_2_, followed by autodetachment from the decarboxylated motor anion which is expected to have a lower binding energy. We have identified CO_2_ loss as a competing decay channel in protein chromophore anions containing COO^–^ using MS-MS measurements.^56^

### 3.2 Electronic structure of the molecular motor

**Fig. 3:**
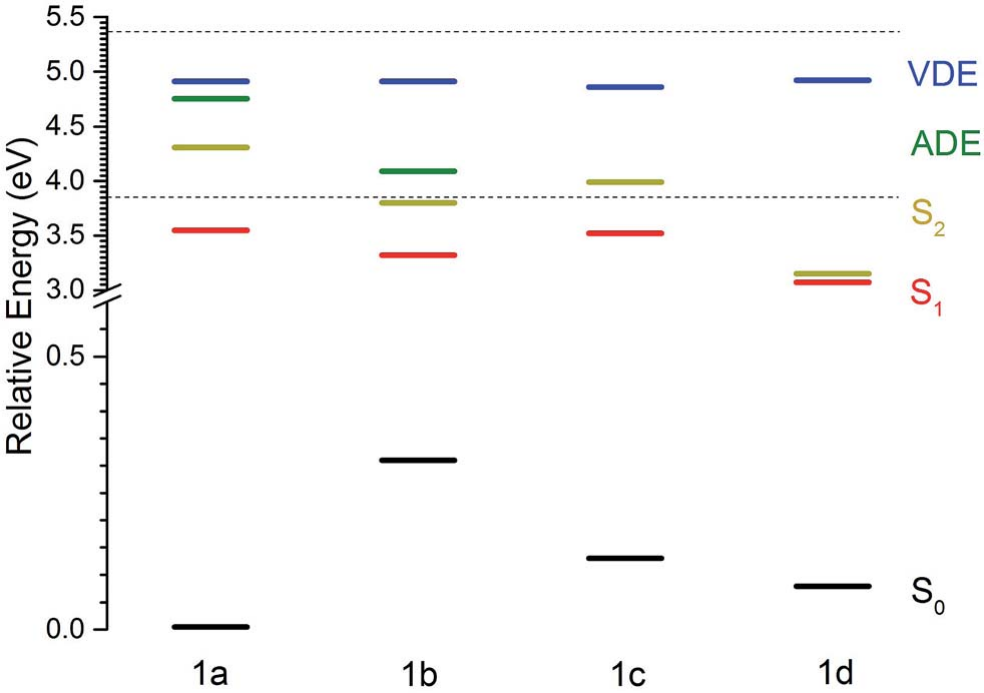
MS-CASPT2 energies of the ground electronic state, S_0_ and the first two singlet excited states, S_1_ and S_2_, of deprotonated molecular motor anions **1a–1d** (R = COO^–^), together with the VDEs and ADEs of the ground electronic state, D_0_, of the corresponding neutral radicals. All energies are in eV and relative to the ground electronic state of deprotonated **1a**. The horizontal dotted lines indicate the minimum and maximum photon energies used in the photoelectron spectroscopy measurements.

The MS-CASPT2 calculated ground-state energies, VEEs, VDEs and ADEs of conformers **1a–1d**, relative to the ground electronic state of conformer **1a**, are listed in Table 1 and plotted in Fig. 3. Notably, although the VDE is more or less constant for all conformers, the ADE is lowered by approximately 0.7 eV for **1b** compared to **1a**.

**Table 1:** MS-CASPT2 energies of the ground electronic state, S_0_, and the first two singlet excited states, S_1_ and S_2_, of deprotonated molecular motor anions **1a–1d** (R = COO^–^), together with the VDEs and some ADEs of the ground electronic state, D_0_, of the corresponding neutral radicals. All energies are in eV and relative to the ground electronic state of **1a**. Values in parentheses are calculated oscillator strengths

Conformer	S_0_	S_1_	S_2_	D_0_ (VDE)	D_0_ (ADE)
**1a**	0.00	3.55 (0.254)	4.31 (0.074)	4.91	4.75
**1b**	0.31	3.32	3.80	4.91	4.09
**1c**	0.13	3.52	3.99	4.86	—
**1d**	0.08	3.07	3.15	4.92	—

The key natural molecular orbitals (NMOs) involved in the photoexcitation and subsequent rotation of the motor from **1a** to **1b**, *i.e.* the NMOs in which there is a significant change in the natural orbital occupation (NOO) between the ground state and the first electronically excited singlet state are listed in Table 2 and in the ESI.† Note that we only consider S_1_ in our discussions below because for the lowest energy **1a** conformer, the oscillator strength of S_2_ is significantly lower than that of S_1_ and the VEE of S_2_ is higher than the photon energies corresponding to 320–298 nm, suggesting that it does not play a significant role in the excited state dynamics following excitation in this range.

**Table 2:** Natural occupation numbers of key natural molecular orbitals for the deprotonated motor anions **1a–1d** (R = COO^–^)

NMO	**1a**			**1b**			**1c**		**1d**	
	S_0_	S_1_	D_0_	S_0_	S_1_	D_0_	S_0_	S_1_	S_0_	S_1_
98	1.894	1.151	1.005	1.947	1.144	1.001	1.939	1.086	1.942	1.026
99	1.879	1.743	1.936	1.872	1.851	1.924	1.892	1.829	1.897	1.886
103	0.132	0.891	0.029	0.133	0.875	0.065	0.113	0.935	0.108	0.990
106	0.104	0.242	0.066	0.065	0.091	0.055	0.065	0.145	0.069	0.097

**Fig. 4:**
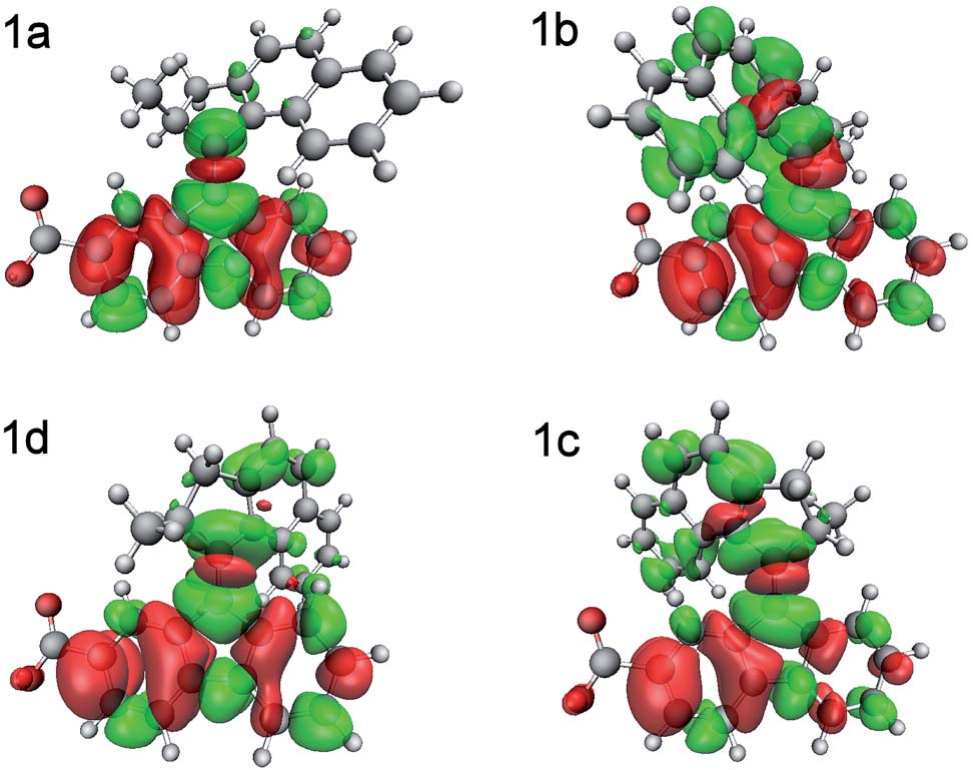
Electron density difference plots between ground state and first excited state of the four conformers of the deprotonated molecular motor **1a–1d** (R = COO^–^). Green areas indicate density accumulation and red areas indicate density depletion.

To illustrate the redistribution of electron density that occurs during the S_0_ → S_1_ transition, the electron density differences between the NMOs involved in this transition are also plotted for each conformer **1a–1d**, in Fig. 4. The initial S_0_ → S_1_ excitation of conformer **1a** has π → π* character on the axle of the motor, consistent with the idea that the initial dynamics are governed by softening of the axle double bond followed by rotation.^22^ This transition also involves considerable change in electron density on the stator, which is consistent with the narrow and well-defined absorption spectra reported for neutral molecular motors based on substituted fluorenes.^22,57^ The characteristics of this charge redistribution are maintained when considering conformers **1b–1c**. In terms of key NMOs, the S_0_ → S_1_ transition is dominated by a transfer of population from NMO 98, which is localised on the stator, to NMO 103, which is also localised on the stator but has significant π* anti-bonding character on the axle double bond (Table 2 and ESI†).

To benchmark our calculations, we have compared the MS-CASPT2 calculated energies of the S_0_ and S_1_ potential energy surfaces at some of the key conformations with those for a related, neutral, fluorene-based molecular motor, 9-(2,4,7-trimethyl-2,3-dihydro-1*H*-inden-1-ylidene)-9*H*-fluorene.^26^ On the S_0_ potential energy surface, conformer **1b** of our molecular motor is 0.31 eV higher in energy than conformer **1a**. This energy difference is slightly larger than the equivalent energy difference calculated for the neutral, fluorene-based molecular motor, using DFT with the B3LYP exchange correlation functional and 6-31G* basis set (0.15 eV).^26^

On the S_1_ potential energy surface, conformer **1b** of our molecular motor is 0.23 eV lower in energy than conformer **1a**. This lowering in energy can be rationalised in terms of the increased delocalisation of electron density across both the rotor and the stator (Fig. 4; this delocalisation is also present in **1c** and **1d**) and is the driving force for the initial rotation around the axle of the molecular motor following S_0_ to S_1_ photoexcitation. The energy difference between the **1a** and **1b** conformers on the S_1_ potential energy surface is almost identical to the equivalent energy difference calculated for the neutral, fluorene-based molecular motor, using SA-REBH&HLYP and TD-BH&HLYP methods.^26^

We also perfomed an unrestricted DFT (B3LYP/6-311G++(2df,2pd)) relaxed scan on the S_0_ potential energy surface by rotating around the double bond and, employing a broken symmetry methodology, found a barrier between **1a** and **1b** with height 2.0 eV, which is substantially higher than that found in the neutral fluorene based molecular motor (1.4 eV).^26^ The increase in relative energy of conformer **1b** compared to **1a** and increased barrier height could be attributed to the increased bulk of the rotor and the COO^–^ substituent on the stator of our molecular motor, but it is worth noting that we only performed a single relaxed scan because we used a large basis set, rather than a multidimensional relaxed scan. We also performed a DFT (B3LYP/6-311G++(2df,2pd)) relaxed scan rotating around the double bond from conformer **1b** to **1c** and found a low (0.2 eV) barrier, which is consistent with this step being a thermal process on the ground electronic state.

The similarity between the S_0_ and S_1_ potential energy landscapes of our deprotonated molecular motor and the related, neutral, fluorene-based molecular motor,^26^ confirms our suggestion that the COO^–^ substituent, included for experimental reasons, does not contribute significantly to the photochemistry of the molecular motor.

## 4 Discussion

**Fig. 5:**
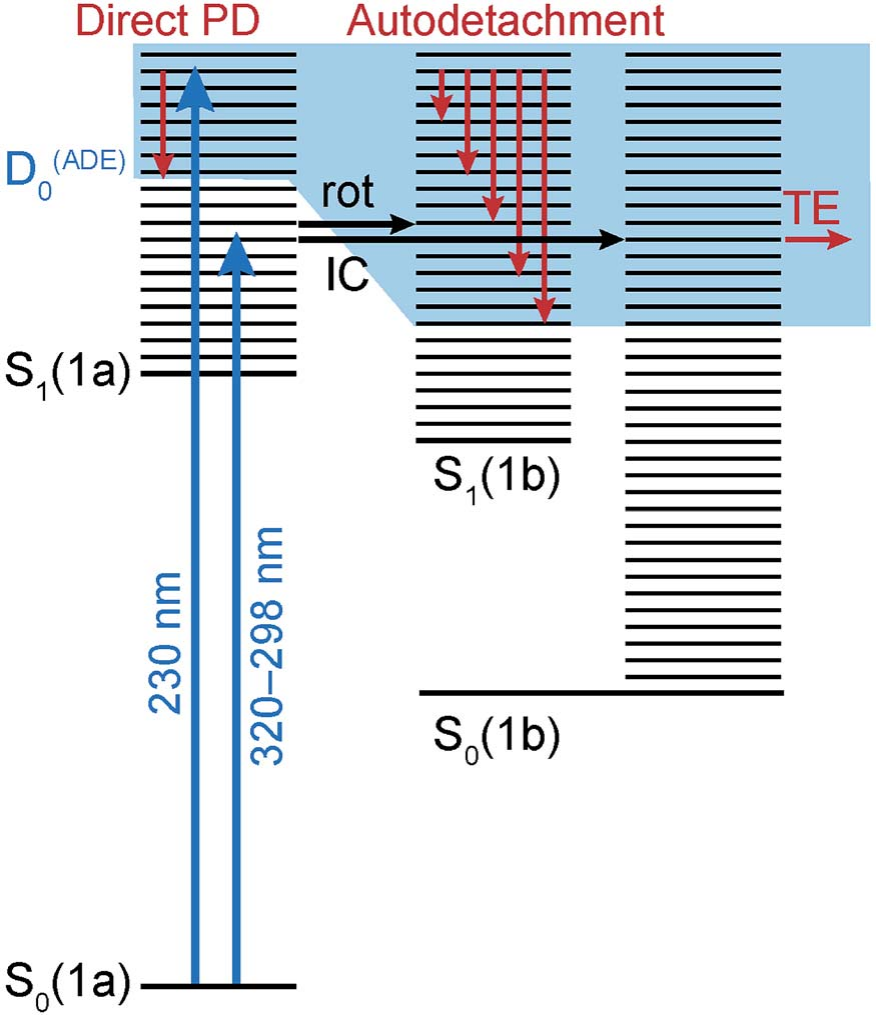
Jablonski diagram illustrating electronic relaxation and electron emission processes following UV photoexcitation of deprotonated **1a** (R = COO^–^). Horizontal black lines represent the vibrational levels of the electronic states and the solid blue area represents the electron detachment continuum. Vertical red arrows represent the eKE of direct and indirect photodetachment (PD) processes. The horizontal black arrows represent internal conversion (IC) to S_0_ and rotation (rot) on S_1_. The horizontal red arrow represents electrons with eKE ∼ 0 following thermionic emission (TE) from vibrationally hot S_0_.

The electronic relaxation and electron emission processes possible following photoexcitation are illustrated on a Jablonski diagram in Fig. 5. The S_1_ state has shape resonance character with respect to the D_0_ continuum, implying a strong coupling between vibrationally excited states of S_1_ that lie above the ADE of the D_0_ continuum.

Following photoexcitation at 320–298 nm, population is transferred from the S_0_ state of conformer **1a** to the S_1_ state of conformer **1a**, below the ADE. Since excitation is below the ADE, direct electron detachment is not possible. The molecule rotates around its axle as it relaxes along the S_1_ potential energy surface. Population then either remains on the S_1_ potential surface or it undergoes internal conversion to S_0_, returning to **1a** or forming the rotated conformer **1b**. Thermionic emission from the ground electronic state of conformer **1a** is not possible because the internal energy is lower than the ADE; however, the ADE of conformer **1b** is approximately 0.7 eV lower than that of conformer **1a**, and thermionic emission from the ground electronic state of conformer **1b** is possible. Thus, we assign the low eKE features in the photoelectron spectra recorded at 320–298 nm to thermionic emission from conformer **1b**. Importantly, this indicates that the initial dynamical steps of the isolated deprotonated rotary motor are similar to those observed for rotary motors in solution, *i.e.* the motor rotates on the excited electronic state and undergoes internal conversion back to the electronic ground state.

**Fig. 6:**
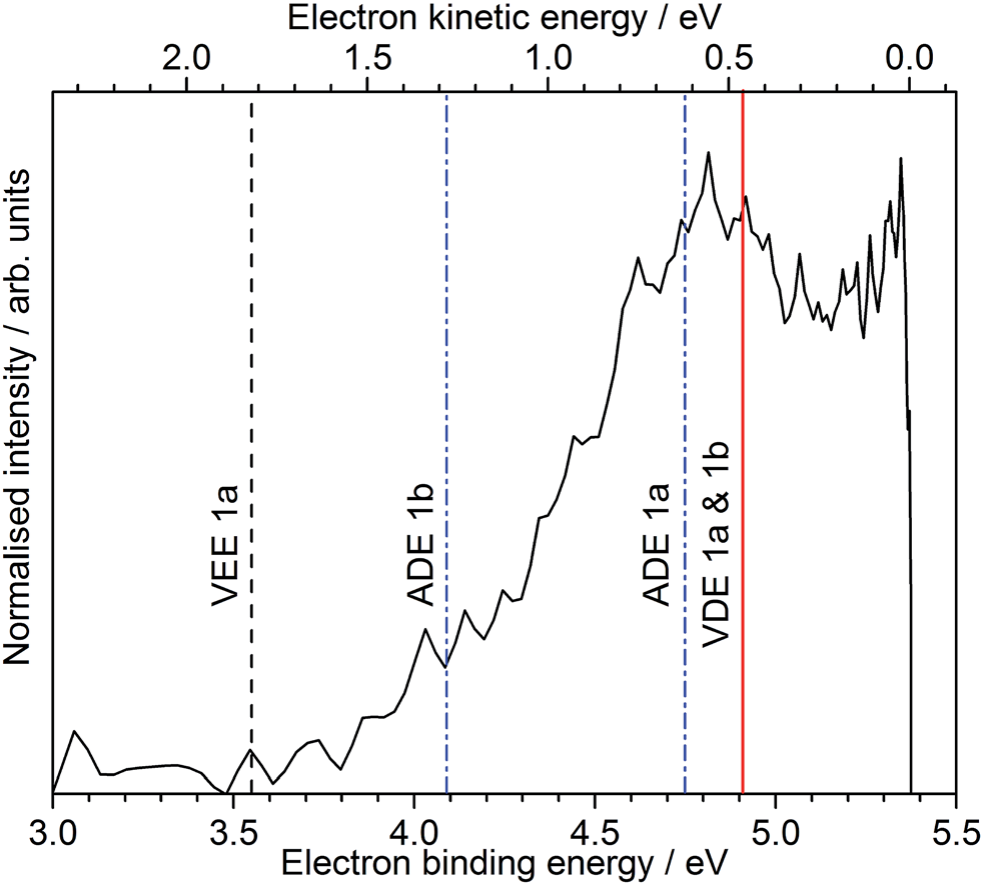
230 nm (5.39 eV) photoelectron spectrum of deprotonated molecular motor **1a** (R = COO^–^), plotted as a function of eBE = *hν* – eKE (bottom axis) and eKE (top axis). The dashed black line marks the MS-CASPT2 calculated VEE of conformer **1a**. The solid red line marks the MS-CASPT2 calculated VDEs of conformers **1a** and **1b** (which are identical) and the dashed blue lines mark the MS-CASPT2 calculated ADEs of conformers **1a** and **1b**.

Following photoexcitation at 230 nm, population is transferred from the S_0_ state of conformer **1a** to the S_1_ state of conformer **1a**, above the ADE. The 230 nm photoelectron spectrum is reproduced in Fig. 6 and plotted as a function of electron binding energy, eBE = *hν* – eKE (bottom axis) and eKE (top axis). The MS-CASPT2 calculated VDEs and ADEs for motor conformers **1a** and **1b** and the VEE for conformer **1a** are also marked on this plot. The calculated VDE for conformer **1a** (4.91 eV) is very close to the maximum of the broad feature of the photoelectron spectrum (4.8 eV), supporting our assignment of this feature to direct detachment to the electronic continuum associated with the ground electronic state of the neutral radical, D_0_. However, the ADE for conformer **1a** is very close to the VDE, so the photoelectrons observed with eBEs lower than the ADE must either result from direct detachment from vibrationally hot S_0_ to the D_0_ continuum associated with the **1a** conformer, or to indirect detachment from S_1_ to D_0_ of the **1b** conformer. Since the low eBE edge of the photoelectron spectrum is very close to the calculated ADE of the **1b** conformer, it seems most likely that these lower eBE photoelectrons arise from autodetachment from the S_1_ state of the **1b** conformer. Importantly, the observation of autodetachment from the S_1_ state of the **1b** conformer as well as thermionic emission is indicative of competing relaxation processes. Thus, we can deduce that following excitation at 230 nm and relaxation on the S_1_ potential energy surface some population remains on the S_1_ potential energy surface and some population undergoes internal conversion back to S_0_. Thermionic emission is possible from both **1a** and **1b** conformers with the 5.39 eV of internal energy in S_0_ that comes from excitation at 230 nm. Our observations are consistent with recent time-resolved studies of second generation molecular rotary motors in solution which observed an initial relaxation out of the Franck–Condon region to a ‘dark state’, which subsequently decays back to the ground electronic state.^22–24^ The similarity between the gas and solution phases suggests that the mechanical motion associated with the initial photoisomerisation step of the rotary cycle is independent of the solvent environment.

## 5 Conclusions

We have shown that the combination of electrospray-ionisation, anion photoelectron spectroscopy and high-level computational chemistry calculations provides a powerful toolkit for gaining insight into the primary events that occur following photoexcitation of a unidirectional molecular motor in the gas-phase, free from solvent effects. Interestingly, the dynamics of the fluorene-based molecular motor anion that is the subject of this gas-phase study are similar to those of fluorene-based molecular motors in solution. That is, the initial dynamics are found to involve relaxation away from the Franck–Condon region to a rotated conformer on the excited state and internal conversion to a rotated conformer on the ground electronic state. It will be interesting to extend our photoelectron spectroscopy studies of rotary molecular motor anions in the gas-phase to the time-domain to measure the timescales of the dynamics and compare them with those obtained for analogous molecular rotary motors in solution. Calculations of the electronic structure and excited state dynamics of molecular rotary motors are non-trivial, so gas-phase studies provide important benchmarks for theory. Improving our fundamental understanding of photoactivated molecular rotary motors in this way is important for the future design of photoactivated nanoscale devices.
