# Supplementary material for: Unravelling the electronic structure and dynamics of an isolated molecular rotary motor in the gas-phase
Source: Chem Sci. 2017 Jun 27;8(9):6141–8. doi: 10.1039/c7sc01997a (PMC5627543; doi:10.1039/c7sc01997a)
Supplement: Supplementary file 1 [file SC-008-C7SC01997A-s001.pdf]

Electronic Supplementary Material (ESI) for Chemical Science.
This journal is © The Royal Society of Chemistry 2017

Supplementary Information

Unravelling the electronic structure and dynamics of an isolated molecular rotary motor in the gas-phase

Reece Beekmeyer,[a] Michael A. Parkes,[a] Luke Ridgewell,[a] Jamie W. Riley,[a] Jiawen Chen,[c] Ben L. Feringa,[c] Andrew Kerridge[b] and Helen H. Fielding *[a]

[a]Department of Chemistry, University College London, 20 Gordon Street, London WC1H 0AJ, UK

[b]Department of Chemistry, Lancaster University, Lancaster, LA1 4YB, UK

[c]Stratingh Institute for Chemistry, University of Groningen, Nijenborgh 4, 9747 AG Groningen, The Netherlands

1 Synthesis

Chemicals were purchased from Acros, Aldrich, Fluka or Merck and were used as received. Solvents for extraction and chromatography were technical grade. All solvents used in reactions were freshly distilled from appropriate drying agents before use. All reactions were performed under inert atmosphere (Ar). Analytical TLC was performed with Merck silica gel 60 F254 plates and visualization was accomplished by UV light. Flash chromatography was carried out using Merck silica gel 60 (230-400 mesh ASTM). Solvents for spectroscopic studies were of spectrophotometric grade (UVASOL Merck). Melting points were taken on a Mettler FP-2 melting point apparatus equipped with a Melttler FP-21 microscope. NMR spectra were recorded on Varian AMX400 (1H: 400 MHz, 13C: 100 MHz). The deuterated solvents were treated with Na_2CO_3, molecular sieves (4 Å) and degassed by argon prior to use. Chemical shifts are denoted in parts per million (ppm) relative to the residual solvent peak. The splitting parameters are designated as follows: s = singlet, d = doublet, t = triplet, q = quartet, m = multiplet, dd = doublet of doublets. High-resolution mass spectrometry (ESIMS) was performed on a LTQ Orbitrap XL spectrometer with ESI ionization. Compounds S1 and S2 were prepared according to literature procedures.[1,2]

Compound S3

Lawesson's reagent (322 mg, 0.8 mmol) was added to a solution of **S1** (79 mg, 0.4 mmol) in toluene (10 mL). The mixture was stirred at 110 °C for 2 h after which the solvent was evaporated. The residue was purified by flash column (SiO_2, pentane:CH_2Cl_2 = 10:1) to obtain the thioketone intermediate as a brown oil. Meanwhile, MnO_2 (175 mg, 2 mmol) was added to a solution of hydrazone **S2** (101 mg, 0.4 mmol) in

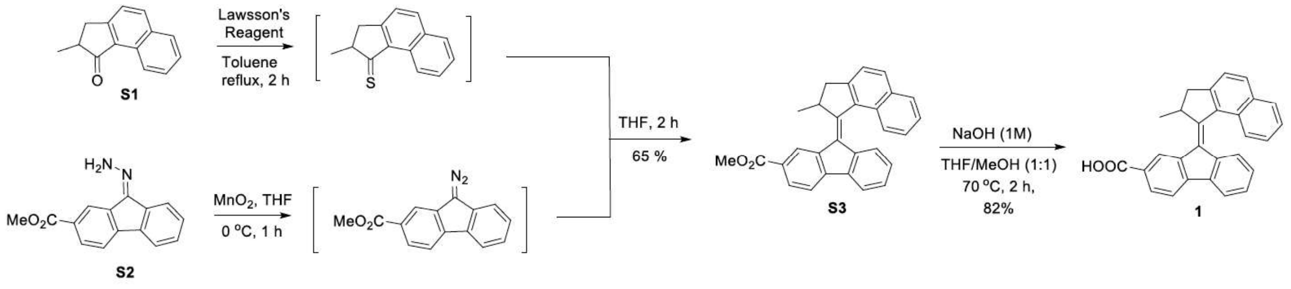

Scheme 1: Synthesis of motor **1**.

THF (15 mL) at 0 °C. After 1 h, the mixture was filtered under N_2, and the obtained pink solution was immediately added to the thioketone intermediate. This mixture was stirred for another 2 h and then concentrated *in vacuo*. The crude residue was purified by column chromatography (SiO_2, pentane:ethyl acetate = 1:3) to give compound **S3** (104 mg, 65%) as an oil. ^{1}H NMR (400 MHz, $CDCl_3$) δ 8.08 8.00 (m, 1H), 8.00 7.86 (m, 3H), 7.85 7.69 (m, 2H), 7.59 (dd, J = 8.2, 2.6 Hz, 1H), 7.53 7.39 (m, 2H), 7.37 7.21 (m, 2H), 6.96 6.74 (m, 2H), 4.41 (dt, J = 22.1, 6.3 Hz, 1H), 4.01 (s, 1H), 3.55 (d, J = 0.6 Hz, 3H), 2.80 (dd, J = 15.2, 5.4 Hz, 1H), 1.47 (dd, J = 16.0, 6.7 Hz, 3H). ^{13}C NMR (101 MHz, $CDCl_3$) δ 166.1, 166.1, 165.8, 147.9, 135.9, 133.2, 132.6, 132.1, 131.9, 131.4, 131.3, 131.1, 130.9, 130.1, 130.0, 129.6, 129.2, 129.1, 128.7, 127.2, 126.8, 126.3, 126.1, 125.4, 125.1, 124.4, 123.9, 84.3, 67.0, 52.4, 52.4, 52.3, 52.2, 41.8, 24.8, 19.4. HRMS (ESI) *m/z*: [M]+ Calcd for $C_{29}H_{22}O_2$ 402.1620, found 402.1633.

Motor 1

Ester **S3** (80 mg, 0.2 mmol) was dissolved in THF (5 mL), MeOH (5 mL) and NaOH(aq.) (1M, 5mL) and heated at 70 °C for 12 h. The mixture was cooled to rt and water (5 mL) was added. THF and MeOH were removed by rotary evaporation. A brown precipitate was formed upon titration of the mixture with HCl(aq.) (1M) until pH=1. After filtration, the brown solid was washed with cold water(aq.) (10 mL) and dried *in vacuo*, affording compound **1** as a solid (67 mg, 82%). M.p. 72 74 °C; ^{1}H NMR (400 MHz, CD3OD) δ 8.74 (s, 1H), 8.14 7.93 (m, 3H), 7.92 7.83 (m, 2H), 7.72 7.55 (m, 2H), 7.52 7.21 (m, 4H), 6.90 6.62 (m, 2H), 4.43 4.34 (m, 1H), 3.62 (dd, J = 15.4, 5.3 Hz, 1H), 2.85 (dd, J = 15.4, 3.7 Hz, 1H), 1.41 (dd, J = 13.8, 6.7 Hz, 3H). ^{13}C NMR (101 MHz, DMSO-d6) δ 164.4, 151.5, 148.1, 139.7, 139.5, 136.1, 132.7, 132.5, 131.6, 129.3, 128.7, 127.7, 127.5, 127.4, 127.0, 126.9, 126.2, 125.8, 125.4, 124.6, 124.2, 122.5, 120.3, 119.7, 53.1, 45.4, 41.9, 19.5. HRMS (ESI) *m/z*: [M]+ Calcd for $C_{28}H_{20}O_2$ 388.1463, found 388.1470.

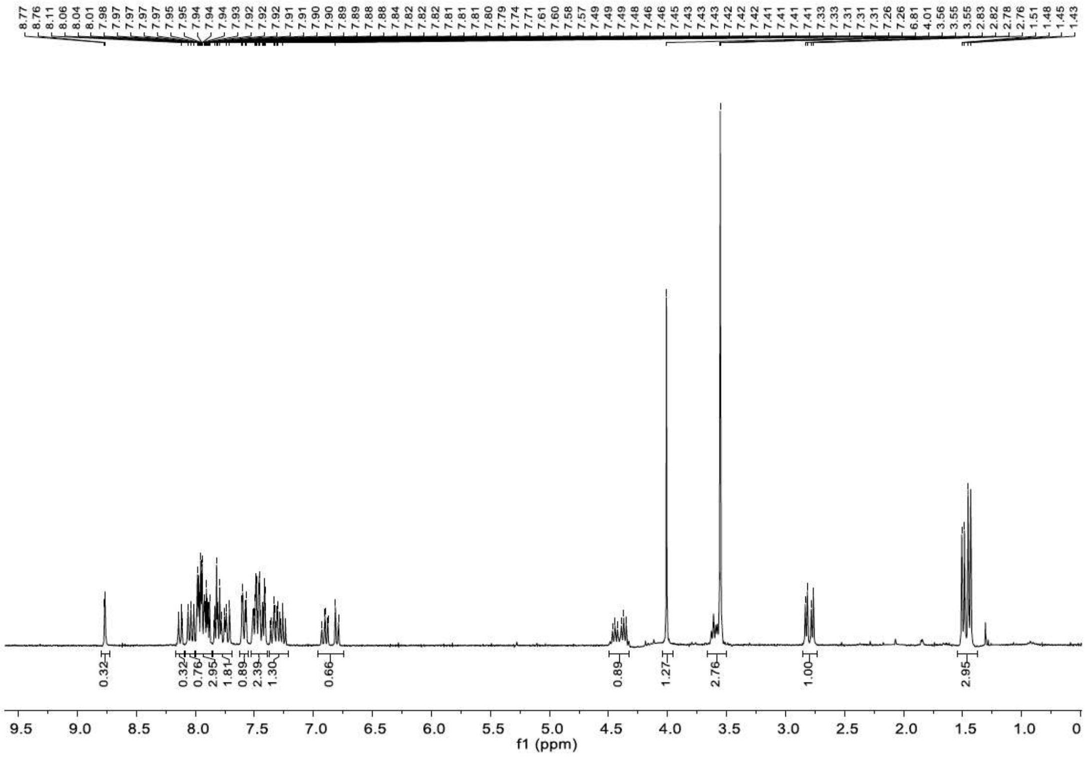

Fig. 1: ^{1}H NMR spectrum of **S3** in $CDCL_3$ at 400 MHz.

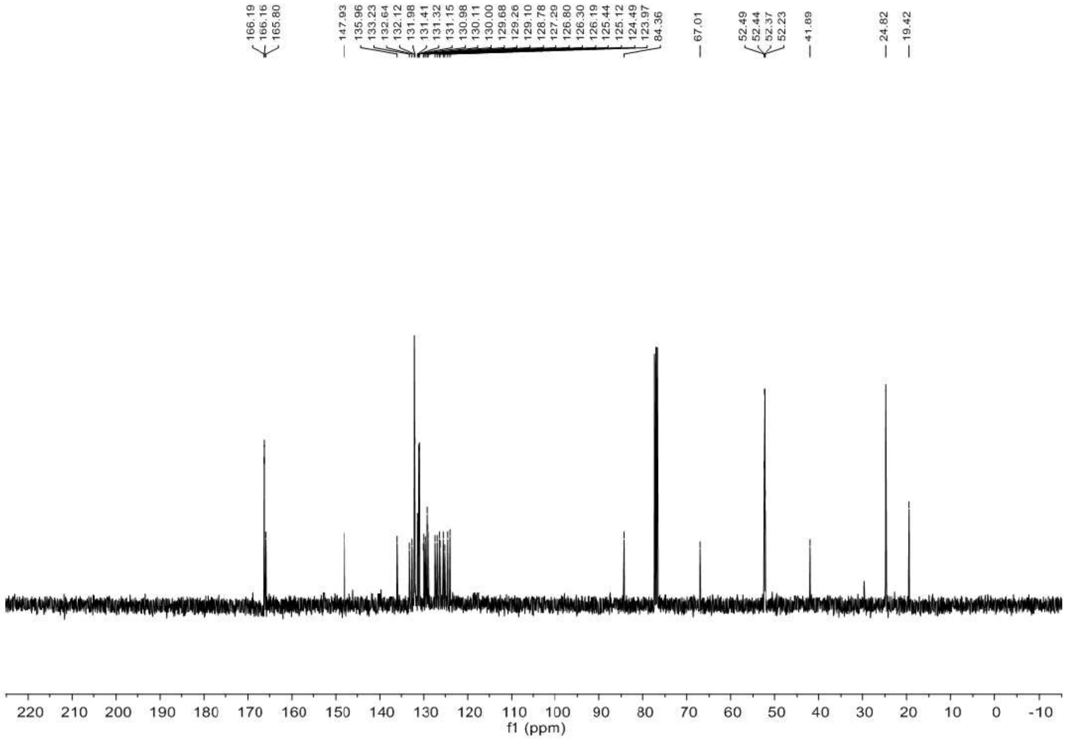

Fig. 2: ^{13}C NMR spectrum of **S3** in $CDCL_3$ at 100 MHz.

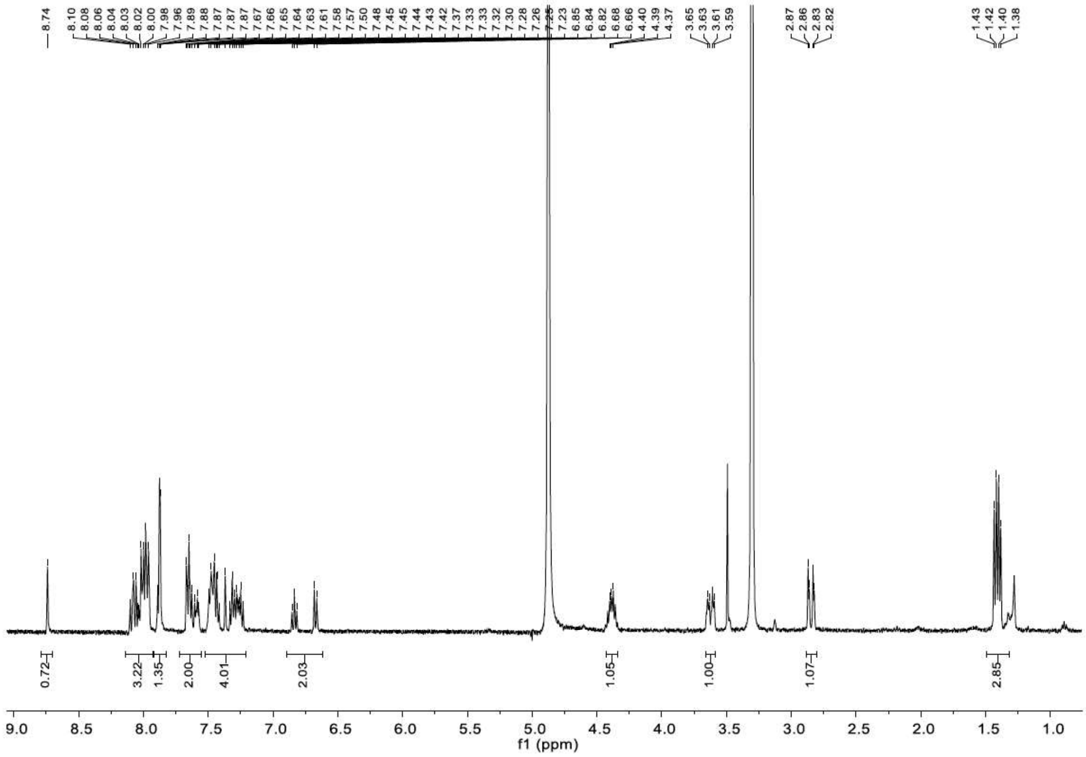

Fig. 3: ^{1}H NMR spectrum of **1** in CD_3OD at 400 MHz.

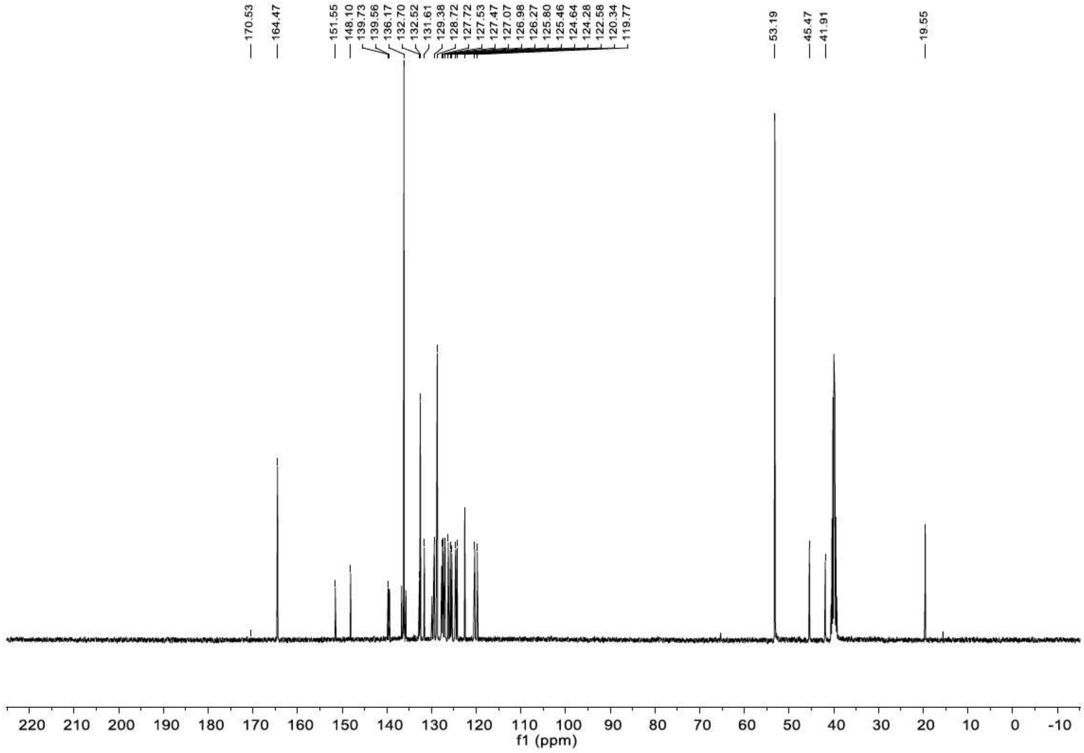

Fig. 4: ^{13}C NMR spectrum of **1** in DMSO-d_6 at 100 MHz.

2 Computational chemistry

Atom	x	y	z
H1	-3.769415	-3.317061	-1.967451
H2	-1.041477	-3.892669	-1.603681
H3	-0.731618	-2.509728	-2.650631
H4	3.938437	3.007079	0.060928
H5	-0.267977	5.477466	-1.086873
H6	1.922379	4.473002	-0.507632
H7	-2.108001	1.619327	-1.001653
H8	-2.256964	4.035894	-1.361565
H9	5.600806	1.169125	0.445940
H10	2.584326	-1.758670	0.203759
H11	-5.565308	-2.062252	-0.825645
H12	-1.548678	0.847467	1.651292
H13	-3.343053	1.884584	2.950378
H14	-5.719047	1.307032	2.500392
H15	-6.267595	-0.393742	0.804841
H16	-0.668415	-3.321226	1.066204
H17	0.661443	-4.171317	0.265140
H18	0.998477	-2.780765	1.286448
H19	1.106971	-2.212901	-1.172756
C1	-1.846679	-1.024657	-0.326994
C2	-3.528914	-2.483621	-1.318905
C3	-2.191355	-2.116083	-1.114056
C4	-0.379348	-0.880570	-0.330425
C5	-0.953119	-2.804829	-1.619989
C6	-0.188310	4.410570	-0.920597
C7	1.040380	3.850092	-0.583562
C8	1.128724	2.478779	-0.375248
C9	-0.017961	1.654026	-0.470063
C10	-1.227804	2.224249	-0.855618
C11	-1.310179	3.599291	-1.071253
C12	2.289635	1.635390	-0.121793
C13	3.623475	1.969954	0.074252
C14	4.551703	0.956509	0.292019
C15	2.816509	-0.708584	0.141134
C16	1.870226	0.286466	-0.100433
C17	0.395760	0.238281	-0.275902
C18	-4.220014	-0.750665	0.232508
C19	-2.855229	-0.366401	0.441262
C20	4.160104	-0.383302	0.328473
C21	5.196931	-1.508939	0.581571
C22	-4.527145	-1.791273	-0.678174
C23	-2.576134	0.585012	1.451987
C24	-3.584282	1.165409	2.178942
C25	-4.931637	0.831856	1.929963
C26	-5.237469	-0.109275	0.982150
C27	0.150355	-2.276443	-0.661987
C28	0.293047	-3.188990	0.565496
O1	6.377521	-1.125766	0.726432
O2	4.725865	-2.668504	0.610320

Table 1: Coordinates in Angstroms of deprotonated molecular motor anion **1a**.

Atom	x	y	z
H1	3.568167	-3.695011	-1.474424
H2	0.732305	-3.264229	-2.624822
H3	0.884335	-4.533872	-1.423294
H4	-5.174581	-2.625427	1.382943
H5	-0.887741	-3.667875	-0.136575
H6	-1.800926	-3.946042	-2.444929
H7	-2.675097	-2.657033	-1.622832
H8	-1.43412	-2.256344	-2.800196
H9	-1.174961	4.706742	-0.547424
H10	-3.189478	3.40753	0.188818
H11	1.091025	1.162503	-0.949651
H12	-4.898633	1.63665	0.993021
H13	-6.211997	-0.388627	1.550208
H14	-2.857691	-2.87515	0.621545
H15	5.066781	-2.230023	-0.161961
H16	0.511613	0.265505	1.765098
H17	2.024694	1.504568	3.21122
H18	4.483446	1.189919	3.036736
H19	5.390951	-0.475828	1.46053
C1	1.219312	-1.595686	-0.149677
C2	3.164722	-2.885283	-0.878842
C3	1.783408	-2.649535	-0.858085
C4	-0.238081	-1.60373	-0.317878
C5	0.731174	-3.459558	-1.547488
C6	-0.607089	-2.967233	-0.932119
C7	-1.702263	-2.95122	-2.002896
C8	-1.143963	3.630719	-0.44389
C9	-2.252591	2.904638	-0.022429
C10	-2.148178	1.523902	0.099606
C11	-0.930175	0.866553	-0.187259
C12	0.152659	1.609551	-0.656728
C13	0.056281	2.995437	-0.778861
C14	-3.147235	0.519544	0.436656
C15	-4.457458	0.654356	0.881753
C16	-5.190536	-0.484002	1.204381
C17	-3.293622	-1.88728	0.662352
C18	-2.561029	-0.762352	0.288203
C19	-1.137479	-0.595581	-0.085826
C20	3.467819	-1.062253	0.689975
C21	2.055771	-0.824199	0.728653
C22	-4.606741	-1.746233	1.106681
C23	3.99467	-2.078661	-0.14418
C24	1.571977	0.099977	1.682133
C25	2.427034	0.795735	2.500132
C26	3.818811	0.611469	2.409027
C27	4.323306	-0.306721	1.527052
C28	1.264635	3.822011	-1.293218
O1	1.102642	5.062354	-1.286916
O2	2.254992	3.149627	-1.654007

Table 2: Coordinates in Angstroms of deprotonated molecular motor anion **1b**.

Atom	x	y	z
H1	-0.647193	-4.357871	-1.794637
H2	-0.185809	-2.919006	-2.703325
H3	-3.296000	-3.464873	-2.075122
H4	6.485345	-0.221823	0.803916
H5	5.028176	1.770729	0.582319
H6	3.136653	-2.798959	0.196614
H7	5.519115	-2.488504	0.606299
H8	-4.945000	-2.117250	-0.822277
H9	3.208811	3.496580	0.145116
H10	1.113226	4.781655	-0.355174
H11	-1.103368	1.209799	-0.740615
H12	-0.652374	0.156508	1.848883
H13	-2.332897	1.299128	3.204460
H14	-4.750673	1.008688	2.723468
H15	-5.470469	-0.509561	0.921487
H16	-0.254008	-4.085302	0.899270
H17	0.996538	-4.996452	0.046121
H18	1.443054	-3.739461	1.206054
H19	1.663867	-2.974951	-1.220405
C1	-1.142868	-1.548439	-0.247175
C2	-2.968070	-2.725666	-1.354603
C3	-1.600288	-2.522291	-1.124368
C4	2.199641	1.596284	0.054347
C5	2.266874	2.982756	-0.009466
C6	1.112945	3.701592	-0.300571
C7	-0.153687	1.659151	-0.497765
C8	0.976804	0.920221	-0.151024
C9	0.331236	-1.565118	-0.245733
C10	-0.443236	-3.288451	-1.705679
C11	5.426957	-0.342864	0.610773
C12	4.610553	0.776997	0.483188
C13	3.254240	0.608633	0.231346
C14	2.690496	-0.684912	0.101401
C15	3.520643	-1.793883	0.254522
C16	4.882923	-1.619354	0.498762
C17	1.221211	-0.543773	-0.096380
C18	-3.470061	-1.057631	0.337351
C19	-2.074497	-0.841316	0.572434
C20	-0.095473	3.051951	-0.566129
C21	-1.359525	3.869332	-0.942780
C22	-3.884015	-1.982579	-0.652211
C23	-1.699808	0.005031	1.641572
C24	-2.644917	0.643306	2.402904
C25	-4.017412	0.473537	2.135088
C26	-4.417534	-0.364032	1.128599
C27	0.704772	-2.975348	-0.707958
C28	0.727846	-4.009083	0.429566
O1	-1.223747	5.111977	-0.895092
O2	-2.362691	3.188848	-1.248973

Table 3: Coordinates in Angstroms of deprotonated molecular motor anion **1c**.

Atom	x	y	z
H1	1.271285	-2.950272	-2.570259
H2	1.234617	-4.092847	-1.239583
H3	4.005230	-3.572825	-1.251541
H4	-0.464729	-2.889314	-0.121260
H5	-1.310969	-3.332642	-2.429704
H6	-2.090756	-1.910868	-1.759455
H7	-0.787155	-1.721265	-2.931495
H8	0.287452	5.356085	-1.501702
H9	-1.875614	4.393822	-0.770540
H10	2.132057	1.501180	-1.270051
H11	2.266770	3.901319	-1.768142
H12	-3.849235	2.955101	0.119910
H13	-5.431810	1.137360	0.809501
H14	-2.406183	-1.781661	0.440578
H15	1.295093	0.961004	1.480822
H16	2.874549	2.145358	2.909718
H17	5.292133	1.560262	2.876887
H18	6.073260	-0.316680	1.481735
H19	5.616379	-2.167701	-0.009420
C1	1.871026	-1.098119	-0.255818
C2	3.678141	-2.664580	-0.760371
C3	2.333104	-2.276247	-0.831405
C4	-0.078417	-2.324764	-0.976289
C5	-1.136700	-2.312062	-2.082147
C6	0.215054	4.300731	-1.271159
C7	-0.998491	3.764253	-0.849598
C8	-1.078390	2.407789	-0.556960
C9	0.064287	1.576709	-0.663603
C10	1.258736	2.120167	-1.128724
C11	1.332215	3.480616	-1.420207
C12	0.431248	-0.952988	-0.498401
C13	1.213182	-3.028075	-1.479411
C14	-2.217944	1.580954	-0.178642
C15	-3.523949	1.921593	0.152000
C16	-4.405688	0.919436	0.546481
C17	-2.679585	-0.744013	0.319258
C18	-1.792026	0.234835	-0.125502
C19	-0.343782	0.173194	-0.411104
C20	4.132896	-0.748130	0.650972
C21	2.756036	-0.352626	0.596401
C22	-3.994008	-0.413382	0.642959
C23	-4.979480	-1.518853	1.103881
C24	4.570923	-1.890345	-0.063460
C25	2.338088	0.692840	1.453061
C26	3.229053	1.357143	2.258976
C27	4.596034	1.018204	2.250161
C28	5.031592	-0.019654	1.468604
O1	-6.136018	-1.127150	1.369919
O2	-4.497894	-2.673247	1.150618

Table 4: Coordinates in Angstroms of deprotonated molecular motor anion **1d**.

Atom	x	y	z
H1	-3.791594	-3.306789	-1.965775
H2	-1.065252	-3.902782	-1.603446
H3	-0.757570	-2.527200	-2.657862
H4	-5.568785	-2.039943	-0.814440
H5	-0.267713	5.463225	-1.098158
H6	1.922755	4.461794	-0.521935
H7	-2.116607	1.609711	-0.998391
H8	-2.253325	4.021241	-1.363400
H9	-1.537536	0.845823	1.666955
H10	-3.328516	1.887126	2.961626
H11	-5.703802	1.318530	2.514486
H12	-6.260296	-0.37893	0.818138
H13	3.917320	3.018966	0.039266
H14	5.592108	1.230549	0.434039
H15	2.549464	-1.75384	0.205509
H16	-0.676614	-3.350365	1.039070
H17	0.666598	-4.181665	0.248753
H18	0.971191	-2.790738	1.289021
H19	1.089751	-2.226916	-1.204204
C1	-1.850570	-1.034924	-0.317848
C2	-3.541806	-2.478778	-1.315209
C3	-2.201592	-2.120783	-1.109251
C4	-0.185495	4.398170	-0.929380
C5	1.040798	3.839401	-0.595970
C6	1.120735	2.467197	-0.385854
C7	-0.020467	1.637799	-0.477848
C8	-1.234014	2.210994	-0.857718
C9	-1.307891	3.582588	-1.073968
C10	-0.385895	-0.896223	-0.337159
C11	-0.972480	-2.816110	-1.624928
C12	2.280421	1.629252	-0.132248
C13	3.612891	1.981417	0.060508
C14	4.549575	0.986521	0.283787
C15	2.805374	-0.709458	0.139646
C16	1.858377	0.275969	-0.107464
C17	0.386447	0.226992	-0.283743
C18	-4.214493	-0.740527	0.244703
C19	-2.848835	-0.363979	0.453738
C20	4.144031	-0.351286	0.323951
C21	5.132935	-1.401783	0.570865
C22	-4.528542	-1.778130	-0.668480
C23	-2.564694	0.586041	1.462438
C24	-3.571831	1.169753	2.189511
C25	-4.919600	0.841471	1.942553
C26	-5.229749	-0.098012	0.994446
C27	0.140691	-2.288919	-0.677313
C28	0.286916	-3.204186	0.549279
O1	6.363169	-1.201217	0.771804
O2	4.878206	-2.636357	0.613757

Table 5: Coordinates in Angstroms of deprotonated molecular motor **1a** in its neutral radical form.

Atom	x	y	z
H1	3.568167	-3.695011	-1.474424
H2	0.732305	-3.264229	-2.624822
H3	0.884335	-4.533872	-1.423294
H4	-5.174581	-2.625427	1.382943
H5	-0.887741	-3.667875	-0.136575
H6	-1.800926	-3.946042	-2.444929
H7	-2.675097	-2.657033	-1.622832
H8	-1.434120	-2.256344	-2.800196
H9	-1.174961	4.706742	-0.547424
H10	-3.189478	3.407530	0.188818
H11	1.091025	1.162503	-0.949651
H12	-4.898633	1.636650	0.993021
H13	-6.211997	-0.388627	1.550208
H14	-2.857691	-2.875150	0.621545
H15	5.066781	-2.230023	-0.161961
H16	0.511613	0.265505	1.765098
H17	2.024694	1.504568	3.211220
H18	4.483446	1.189919	3.036736
H19	5.390951	-0.475828	1.460530
C1	1.219312	-1.595686	-0.149677
C2	3.164722	-2.885283	-0.878842
C3	1.783408	-2.649535	-0.858085
C4	-0.238081	-1.603730	-0.317878
C5	0.731174	-3.459558	-1.547488
C6	-0.607089	-2.967233	-0.932119
C7	-1.702263	-2.951220	-2.002896
C8	-1.143963	3.630719	-0.443890
C9	-2.252591	2.904638	-0.022429
C10	-2.148178	1.523902	0.099606
C11	-0.930175	0.866553	-0.187259
C12	0.152659	1.609551	-0.656728
C13	0.056281	2.995437	-0.778861
C14	-3.147235	0.519544	0.436656
C15	-4.457458	0.654356	0.881753
C16	-5.190536	-0.484002	1.204381
C17	-3.293622	-1.887280	0.662352
C18	-2.561029	-0.762352	0.288203
C19	-1.137479	-0.595581	-0.085826
C20	3.467819	-1.062253	0.689975
C21	2.055771	-0.824199	0.728653
C22	-4.606741	-1.746233	1.106681
C23	3.994670	-2.078661	-0.144180
C24	1.571977	0.099977	1.682133
C25	2.427034	0.795735	2.500132
C26	3.818811	0.611469	2.409027
C27	4.323306	-0.306721	1.527052
C28	1.264635	3.822011	-1.293218
O1	1.102642	5.062354	-1.286916
O2	2.254992	3.149627	-1.654007

Table 6: Coordinates in Angstroms of deprotonated molecular motor **1b** in its neutral radical form.

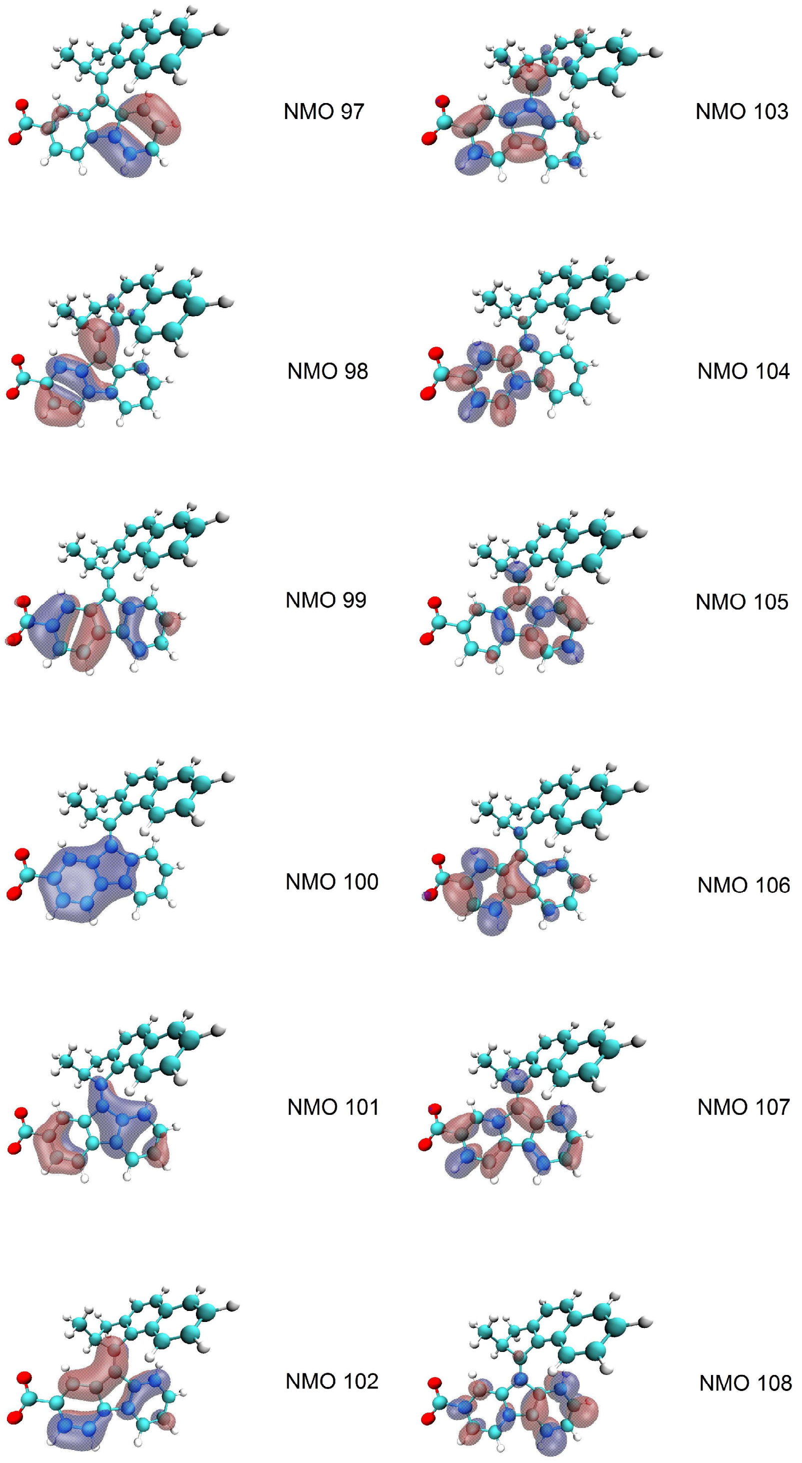

Fig. 5: Natural orbitals comprising the 12 electron, 12 orbital active space used in state-averaged CASSCF calculations of the deprotonated molecular motor anion **1a**.

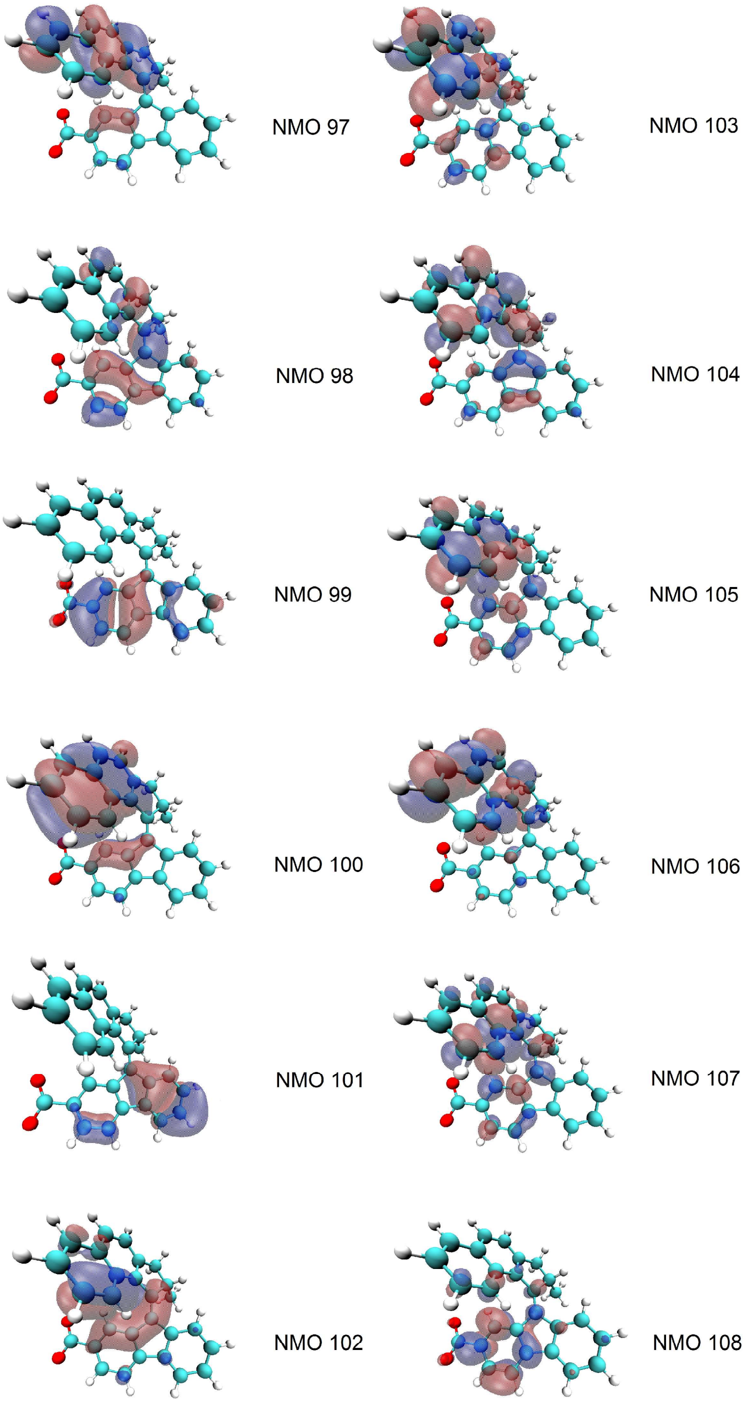

Fig. 6: Natural orbitals comprising the 12 electron, 12 orbital active space used in state-averaged CASSCF calculations of the deprotonated molecular motor anion **1b**.

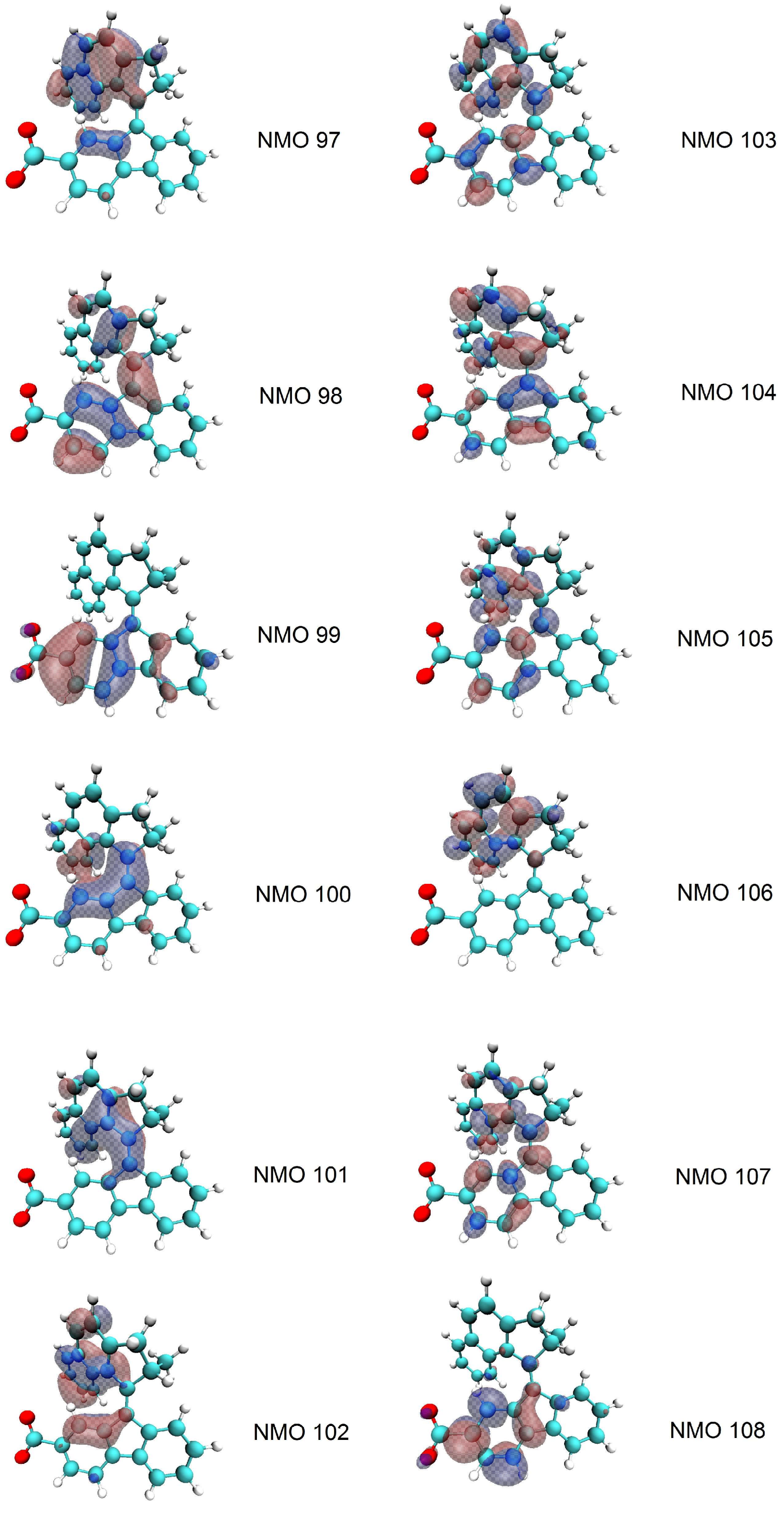

Fig. 7: Natural orbitals comprising the 12 electron, 12 orbital active space used in state-averaged CASSCF calculations of the deprotonated molecular motor anion **1c**.

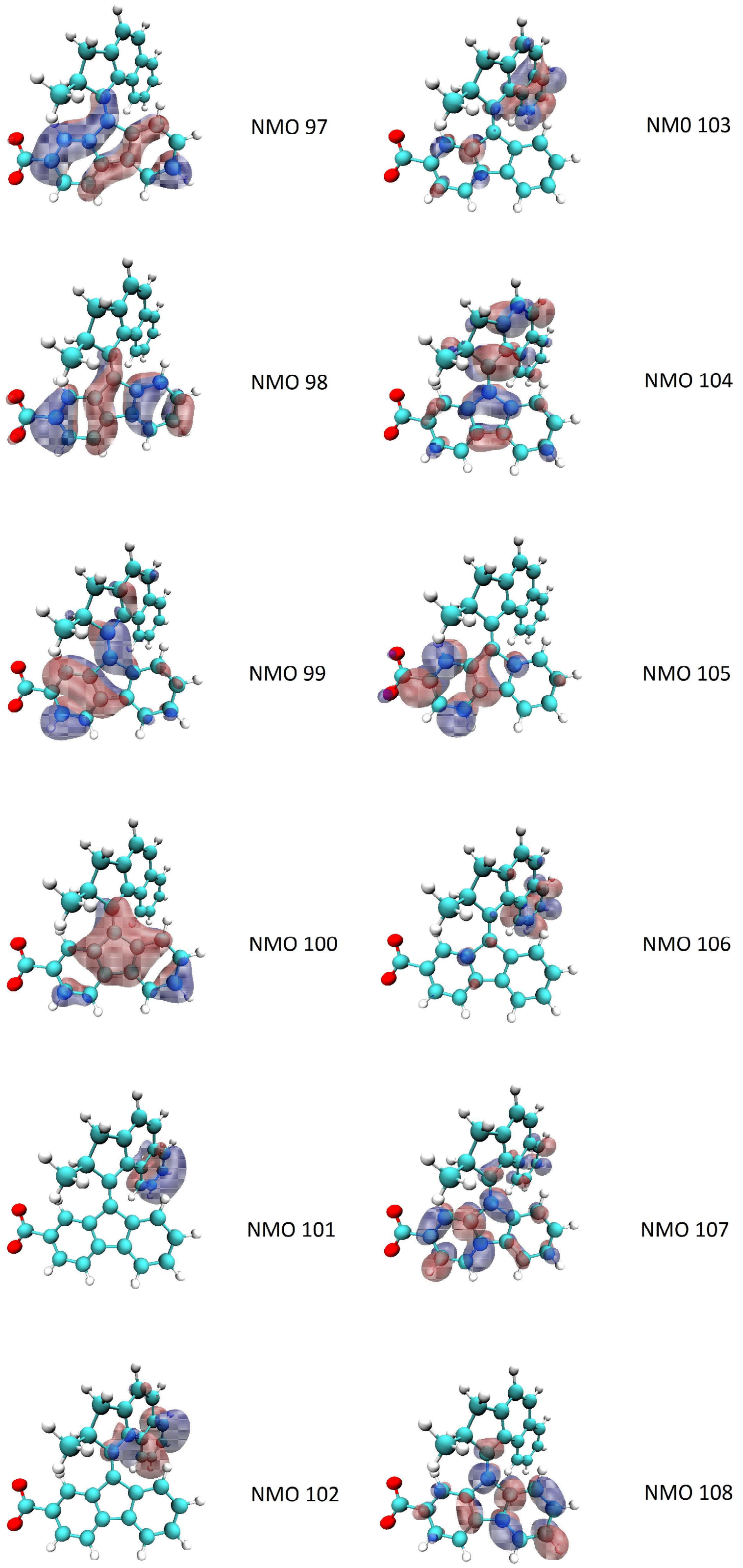

Fig. 8: Natural orbitals comprising the 12 electron, 12 orbital active space used in state-averaged CASSCF calculations of the deprotonated molecular motor anion **1d**.

3 References

[1] J. Vicario, M. Walko, A. Meetsma and B. L. Feringa, *J. Am. Chem. Soc.*, 2006, **128**, 5127–5135.

[2] A. Moritomo, H. Yamada, T. Watanabe, H. Itahana, Y. Koga, S. Akuzawa and M. Okada, *Bioorganic Med. Chem.*, 2014, **22**, 4323–4337.
